# Increased mean corpuscular hemoglobin concentration: A case report as an early clue of chronic severe hyponatremia

**DOI:** 10.1097/MD.0000000000050997

**Published:** 2026-10-09

**Authors:** Jianping Chen, Shi Chen, Xinzheng Li, Baobing Chen, Jiyun Tian

**Affiliations:** a Department of Clinical Laboratory, Hangzhou Third People’s Hospital, Hangzhou, Zhejiang, China.

**Keywords:** cell volume regulation, electrolyte imbalance, hyponatremia, laboratory early clue, mean corpuscular hemoglobin concentration

## Abstract

**Rationale:**

Mean corpuscular hemoglobin concentration (MCHC) is a stable parameter, and its true elevation typically indicates hematological disorders. Although occasional reports suggest an association with electrolyte imbalances, the underlying mechanisms remain unclear.

**Patient concerns:**

An 81-year-old female was admitted with a 3-day history of altered mental status. Complete blood count revealed a markedly elevated MCHC (369 g/L), and peripheral blood smear showed reduced central pallor and mildly hyperchromic erythrocytes, suggestive of cellular dehydration.

**Diagnoses:**

Chronic severe hypotonic hyponatremia (serum sodium 102 mmol/L), initially alerted by a confirmed “true” MCHC elevation after systematically ruling out common analytical interferences.

**Interventions:**

Laboratory personnel issued an early warning for severe electrolyte imbalance prior to biochemical results. The patient subsequently received targeted sodium replacement therapy.

**Outcomes:**

Following sodium replacement, the patient’s serum sodium levels normalized (142 mmol/L), her mental status markedly improved, and her MCHC demonstrated a mirroring downward trend back to normal (337 g/L).

**Lessons:**

Laboratory physicians should consider true MCHC elevation, after excluding interferences, as a potential indicator of chronic hyponatremia. Proactive communication of this finding to clinicians can expedite diagnosis and treatment of critical electrolyte disorders.

## 1. Introduction

The mean corpuscular hemoglobin concentration (MCHC), a parameter calculated by hematology analyzers from hemoglobin (Hb) and hematocrit, is generally stable. Its primary clinical utility lies in monitoring the stability of the analytical system and, to a lesser extent, providing clues for differentiating causes of anemia; for instance, MCHC is often elevated in hereditary spherocytosis. Importantly, abnormal MCHC results can be influenced by various pre-analytical factors, such as in vitro hemolysis, lipemia, and cold agglutination, which may lead to falsely increased values.^[1]^

Hyponatremia, defined as a serum sodium concentration below 135 mmol/L, ranks among the most prevalent electrolyte disorders in clinical practice. Severe hyponatremia (serum sodium <125 mmol/L) can lead to serious neurological complications, including cerebral edema, seizures, coma, and even death.^[2,3]^ While diagnosis relies on biochemical testing, the unavoidable time delay from sample collection to result reporting may hinder timely intervention.^[2]^ This article presents the case of an elderly female admitted with impaired consciousness. Notably, an abnormally elevated MCHC in her complete blood count (CBC) provided an early indication of potentially life-threatening severe hyponatremia before the biochemical results were available. This early clue facilitated targeted treatment, demonstrating the potential utility of MCHC as a rapid screening parameter.

## 2. Case presentation

An 81-year-old female patient was admitted to our hospital on December 29, 2025, due to “altered consciousness and abnormal speech and behavior for three days.” Her past medical history was notable for hypertension, currently managed with oral amlodipine besylate (5 mg once daily); however, her exact blood pressure control status prior to admission was unknown. Furthermore, she had no previous history of diabetes or renal disease and no documented history of dehydration or diuretic use. Physical examination revealed stable vital signs and confusion. However, a comprehensive neurological examination could not be completed due to the patient’s uncooperative state.

### 2.1. Key laboratory findings and clinical intervention

The admission CBC, performed on a Mindray BC-6900 hematology analyzer (Shenzhen Mindray Bio-Medical Electronics Co., Ltd., Shenzhen, China), revealed the following: a white blood cell count of 7.1 × 10^9^/L with 81.1% neutrophils, suggesting possible infection, and an Hb level of 92 g/L, red blood cell count of 2.96 × 10^12^/L, and hematocrit of 0.257, indicating moderate anemia. A critical finding was a mean corpuscular volume (MCV) at the lower limit of normal (84.1 fL) coupled with a markedly elevated MCHC of 369 g/L (reference range: 316–354 g/L).

Upon reviewing these results, the laboratory physician immediately initiated an interference workup. While no visible hemolysis, icterus, or lipemia was noted in the specimen, and the analyzer showed no flags for hemolysis, lipemic interference, or cold agglutination, the sample was incubated at 37°C for 30 minutes and immediately reanalyzed as per standard protocol. The post-incubation MCHC result was 368 g/L, virtually unchanged, thereby excluding cold agglutination as a cause. With daily internal quality control within acceptable limits, the elevated MCHC was considered a true finding.

The laboratory physician reviewing the results recognized that a markedly elevated MCHC is often associated with cellular dehydration secondary to altered extracellular osmolality. Proactively, and prior to the return of the patient’s biochemical electrolyte results – which typically requires over 60 minutes – he immediately telephoned the clinical team to warn that the significant MCHC elevation indicated a potential severe electrolyte imbalance. Concurrently, within the laboratory, he coordinated to expedite processing of the patient’s biochemical specimen.

Subsequent biochemical results (Hitachi 7180 Automatic Analyzer, Hitachi High-Tech Corporation, Tokyo, Japan) confirmed this preliminary warning: the patient exhibited severe hyponatremia (serum sodium: 102 mmol/L), with accompanying hypokalemia (3.23 mmol/L) and hypochloremia (67 mmol/L). The calculated serum osmolality was approximately 254.4 mOsm/kg H_2_O, consistent with hypotonic hyponatremia. Other findings included a urea nitrogen level of 6.00 mmol/L, creatinine of 70 μmol/L, and C-reactive protein of 0.3 mg/L. Thyroid and liver function tests were largely unremarkable. Imaging studies of the head and lungs revealed age-related cerebral changes and mild inflammatory signs.

### 2.2. Clinical management and patient outcome

Based on the patient’s history, laboratory findings, and imaging studies, a clinical diagnosis of severe hyponatremia (hypotonic) and community-acquired pneumonia was established. During treatment, we dynamically monitored serum sodium and MCHC levels at specified time points (Fig. 1). As illustrated in Figure 1, serum sodium progressively increased from 102 mmol/L at admission (T0, pretreatment) through subsequent monitoring intervals (T1, T2…), a trend accompanied by a corresponding improvement in the patient’s mental status. Notably, MCHC levels demonstrated a near-mirror-image decline in relation to serum sodium during this period. The patient received staged intravenous fluid replacement therapy (detailed protocol provided in Table S1, Supplemental Digital Content 1). When serum sodium was corrected to within the normal range (approximately 137 mmol/L), the MCHC concurrently returned to a normal level of 337 g/L. The patient’s mental status improved progressively in parallel with the correction of serum sodium. After the electrolyte imbalance was resolved, she completed a course of antibiotic therapy for community-acquired pneumonia. Her condition remained stable without recurrence of hyponatremia, and she was discharged in good clinical condition on January 4, 2026.

**Figure 1.**
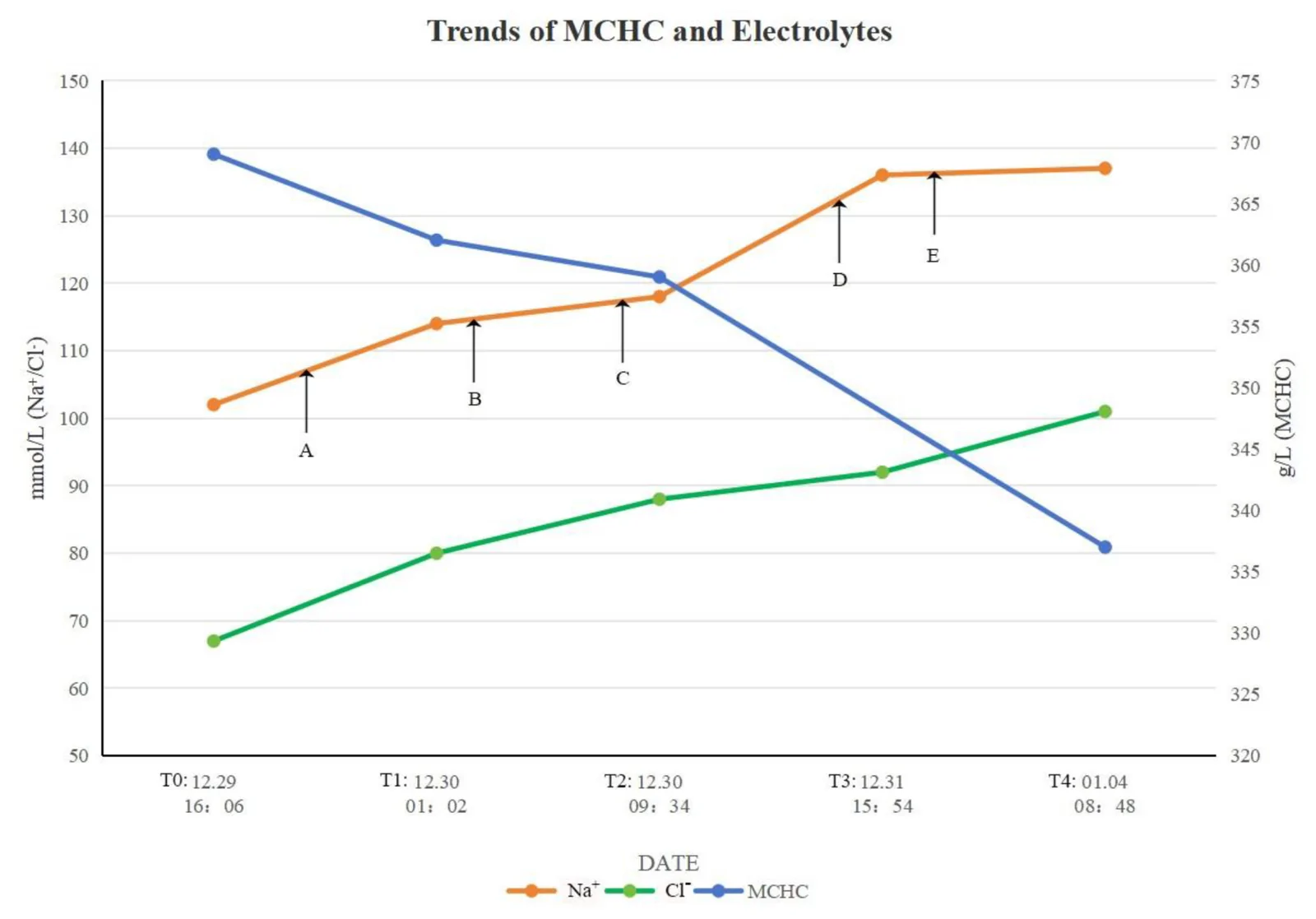
Dynamic monitoring of serum sodium and MCHC during treatment. Data points correspond to laboratory measurement time points. Following admission, the patient received staged intravenous sodium and potassium replacement therapy. Specifically, letters A to E indicate the exact start time points of intravenous fluid therapy: A (December 29, 20:08), B (December 30, 02:48), C (December 30, 08:08), D (December 30, 16:08), and E (January 1, 08:56). The detailed fluid compositions are provided in Table S1, Supplemental Digital Content 1. As illustrated, the serum sodium concentration progressively increased with therapeutic intervention, while the MCHC exhibited a near-mirror-image decline. The 2 parameters demonstrated a significant inverse correlation throughout the treatment course. MCHC = mean corpuscular hemoglobin concentration.

### 2.3. Peripheral blood smear findings

The peripheral blood smear at admission (Wright-Giemsa stain, ×1000) revealed that, despite a normal MCV, a subset of erythrocytes exhibited reduced central pallor with a mildly hyperchromic appearance (Fig. 2A). Occasional crenated cells were observed, while spherocytes, schistocytes, or significant polychromasia were absent. Following correction of hyponatremia, a repeat smear showed restoration of normal erythrocyte morphology, characterized by reappearance of distinct central pallor and resolution of the hyperchromic appearance (Fig. 2B).

**Figure 2.**
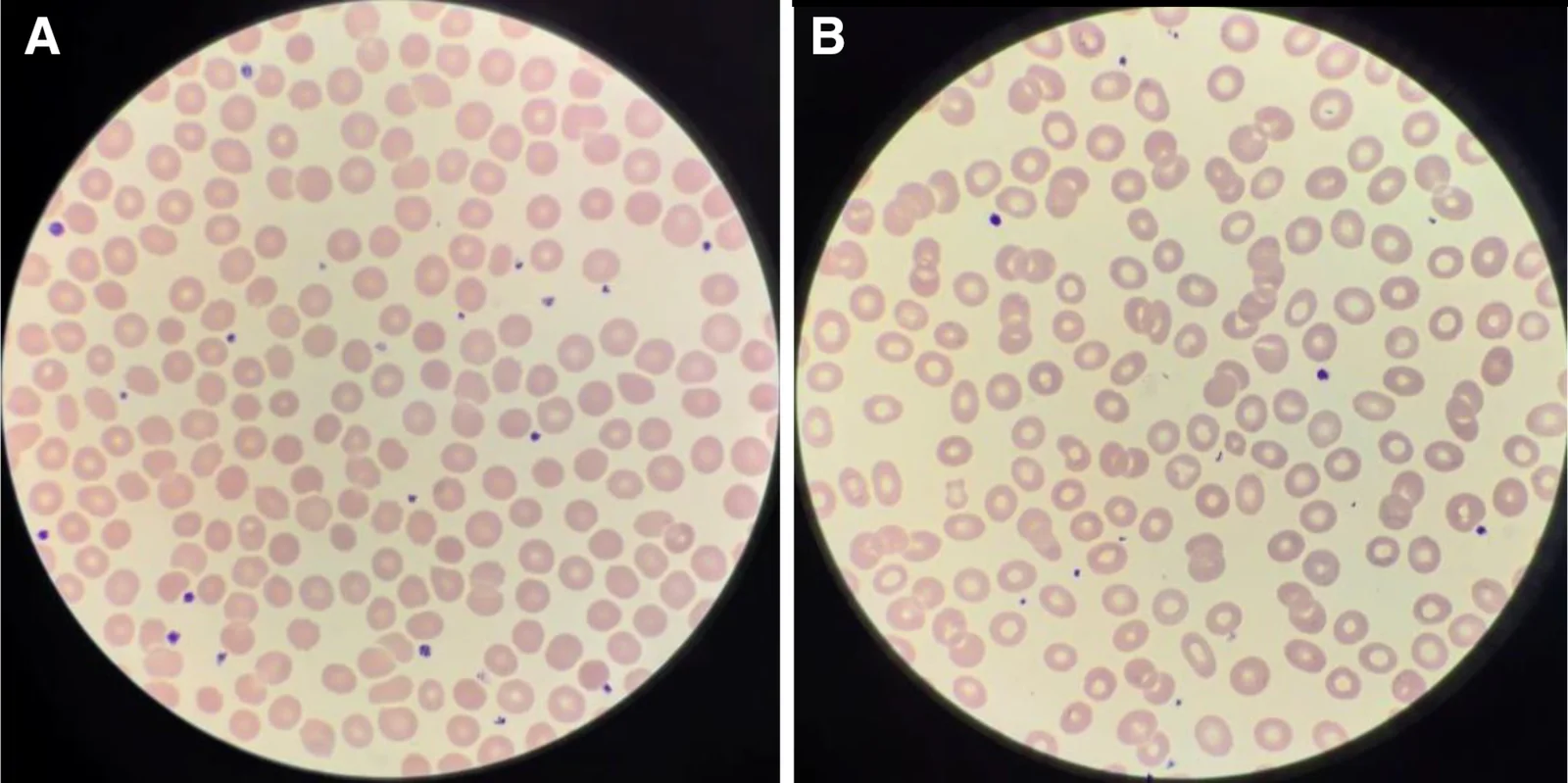
Peripheral blood smear (Wright-Giemsa stain, ×1000) before and after correction of hyponatremia in the patient. (A) Pretreatment (serum sodium: 102 mmol/L, MCHC: 369 g/L): Overall erythrocyte morphology was largely preserved; however, some cells exhibited mild crenation with reduced central pallor, presenting a hyperchromic appearance. (B) Posttreatment (serum sodium: 137 mmol/L, MCHC: 337 g/L): Erythrocyte morphology returned to normal, characterized by the reappearance of appropriately sized central pallor and the disappearance of the hyperchromic appearance. MCHC = mean corpuscular hemoglobin concentration.

## 3. Discussion

This case highlights a clinically significant pattern observed in patients with chronic severe hyponatremia: a “paradoxical” CBC profile characterized by a normal MCV alongside a markedly elevated MCHC, with corresponding morphological evidence of erythrocyte dehydration on peripheral blood smear. This distinct hematological signature may serve as an early laboratory clue prior to the availability of confirmatory biochemical results.

Classical physiology posits that in acute hypotonic conditions, water moves along the osmotic gradient into cells, causing cellular swelling. In red blood cells, this manifests as an increased MCV and a dilutional decrease in the MCHC.^[4]^ However, the present case – characterized by severe hyposmolality alongside a normal-to-low MCV and an elevated MCHC – exhibits a “paradoxical” profile consistent with chronic hyponatremia. The underlying mechanism involves the activation of a volume-regulatory adaptation in erythrocytes, known as regulatory volume decrease.^[5,6]^

Research indicates that under chronic hyposmotic stress (typically >48 hours), mechanosensitive cation channels, notably PIEZO1, play a crucial role in various cell types, including red blood cells. Cell swelling in a hypotonic environment increases membrane tension, activating PIEZO1 and triggering calcium influx. This, in turn, activates downstream pathways that actively export intracellular ions such as K^+^ and Cl^−^, along with organic osmolytes such as myo-inositol and taurine.^[5]^ By reducing the total intracellular solute content, this process actively lowers intracellular osmolality to match the external hypotonic environment, thereby preventing continuous swelling and eventual lysis. Crucially, Hb – a major macromolecular protein within erythrocytes – cannot be extruded and remains relatively constant in quantity. As the cell volume normalizes or even slightly decreases due to the loss of solutes and water, the Hb concentration per unit volume inevitably rises, resulting in a “true” elevation of MCHC in laboratory testing. In this case, the normalization of serum sodium and the restoration of extracellular osmolality following treatment allowed erythrocytes to undergo reverse regulation, re-accumulating solutes and water. The concomitant return of MCHC to normal levels further demonstrates the reversible nature of this adaptive mechanism.

The morphological findings from this case provide direct visual evidence supporting the volume-regulatory mechanism. The hyperchromic appearance with reduced central pallor observed at admission is the characteristic light-microscopic correlate of erythrocyte dehydration – the morphological endpoint of regulatory volume decrease under chronic hypotonic stress. The reversible normalization of erythrocyte morphology following sodium correction parallels the dynamic changes in MCHC and serum sodium (Fig. 1) and offers intuitive, morphology-based reinforcement of the adaptive volume regulation theory.

In emergency and critical care medicine, time is of the essence. This case demonstrates a practical, rapid screening clue for early detection of critical electrolyte imbalances. A CBC, providing results within minutes, proved far quicker than standard biochemical electrolyte testing. Here, recognizing the clinical significance of a sharply elevated MCHC, the laboratory physician followed standardized procedures to exclude pre-analytical interference. The resulting early warning was communicated approximately 50 minutes before critical biochemical results were available. Leveraging this simple routine parameter allowed the clinical team to anticipate and initiate management of severe electrolyte derangement promptly. This approach underscores the essential consultative role of modern laboratory medicine in patient care.

It is essential to emphasize that an elevated MCHC is not a specific biomarker for hyponatremia. Before issuing any clinical alert, more common causes of a falsely high MCHC – as systematically ruled out in this case – and primary hematologic disorders, such as spherocytosis, must be rigorously excluded. Consequently, its clinical utility lies more in serving as a highly specific indicator – once pre-analytical and hematologic confounders are eliminated – with likely limited sensitivity. It functions primarily as a suggestive or triggering signal, prompting further diagnostic investigation.

This pattern alerts both laboratory professionals and clinicians that an unexplained elevation in MCHC should prioritize consideration of severe electrolyte disturbances, particularly chronic hyponatremia. The present study has certain limitations, including the lack of directly measured serum osmolality as definitive evidence of hypotonicity, reliance on calculated values, and an incompletely elucidated etiology for the coexisting anemia. Nevertheless, the strong temporal correlation observed between the correction of hyponatremia and the normalization of MCHC throughout the clinical course remains clear and compelling.

## 4. Conclusion

This case report demonstrates that, after rigorously excluding analytical interference, a markedly elevated MCHC in a routine CBC may serve as an early and valuable laboratory clue of chronic, severe hyponatremia. This sign is rooted in the adaptive volume-regulatory response of red blood cells to a chronic hypotonic environment. Laboratory physicians, by understanding the underlying pathophysiology and maintaining vigilance during result review, can enhance their clinical impact. Proactive communication with the clinical team allows them to translate this laboratory finding into actionable insight, thereby actively contributing to the rapid diagnostic process for critically ill patients and ultimately improving clinical outcomes.

## Author contributions

**Conceptualization:** Jianping Chen, Jiyun Tian.

**Formal analysis:** Shi Chen.

**Data curation:** Xinzheng Li, Baobing Chen.

**Methodology:** Jianping Chen.

**Project administration:** Xinzheng Li.

**Resources:** Xinzheng Li.

**Writing – original draft:** Jianping Chen, Jiyun Tian.

**Writing – review & editing:** Jiyun Tian.


