# Supplementary material for: Increased mean corpuscular hemoglobin concentration: A case report as an early clue of chronic severe hyponatremia
Source: Medicine (Baltimore). 2026 Oct 9;105(41):e50997. doi: 10.1097/MD.0000000000050997 (PMC13657595; doi:10.1097/MD.0000000000050997)
Supplement: Supplementary file 1 [file medi-105-e50997-s001.docx]

Supplementary table 1: Timeline and composition of intravenous fluid therapy administered for hyponatremia correction.

| Time Point | Approximate Start Time | Intravenous Fluid Regimen |
| --- | --- | --- |
| A | 2025/12/29 20:08 | 0.9% Sodium Chloride 500 mL + 10% Sodium Chloride 30 mL |
|  |  | 0.9% Sodium Chloride 500 mL + 10% Sodium Chloride 20 mL |
|  |  | 0.9% Sodium Chloride 500 mL + 10% Sodium Chloride 30 mL |
| B | 2025/12/30 2:48 | Glucose in Sodium Chloride Injection 250 mL + 10% Potassium Chloride 7.5 mL |
|  |  | 0.9% Sodium Chloride 250 mL |
| C | 2025/12/30 8:08 | 0.9% Sodium Chloride 500 mL + 10% Potassium Chloride 10 mL + 10% Sodium Chloride 30 mL |
|  |  | 0.9% Compound Sodium Chloride (Ringer's Solution) 500 mL |
| D | 2025/12/30 16:08 | 0.9% Sodium Chloride 250 mL + 10% Sodium Chloride 30 mL |
| E | 2026/1/1 8:56 | 0.9% Sodium Chloride 250 mL |

Note: This regimen was administered under close monitoring of serum sodium levels and neurological status to avoid overcorrection. The laboratory monitoring time points are shown in Figure 1 of the main text.
